# Hypothesizing an algorithm from one example: the role of specificity

**DOI:** 10.1098/rsta.2022.0046

**Published:** 2023-07-24

**Authors:** S. H. Muggleton FREng

**Affiliations:** Department of Computing, Imperial College London, London, UK

**Keywords:** program synthesis, Bayesian machine learning, logic learning

## Abstract

Statistical machine learning usually achieves high-accuracy models by employing tens of thousands of examples. By contrast, both children and adult humans typically learn new concepts from either one or a small number of instances. The high data efficiency of human learning is not easily explained in terms of standard formal frameworks for machine learning, including Gold’s learning-in-the-limit framework and Valiant’s probably approximately correct (PAC) model. This paper explores ways in which this apparent disparity between human and machine learning can be reconciled by considering algorithms involving a preference for specificity combined with program minimality. It is shown how this can be efficiently enacted using hierarchical search based on identification of certificates and push-down automata to support hypothesizing compactly expressed maximal efficiency algorithms. Early results of a new system called DeepLog indicate that such approaches can support efficient top-down construction of relatively complex logic programs from a single example.

This article is part of a discussion meeting issue ‘Cognitive artificial intelligence’.

## 1. Introduction

**Figure 1.**
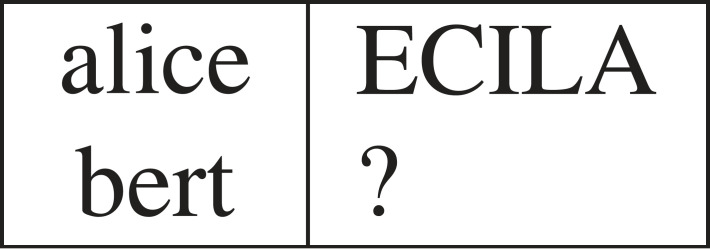
Analogy problem.

**Figure 2.**
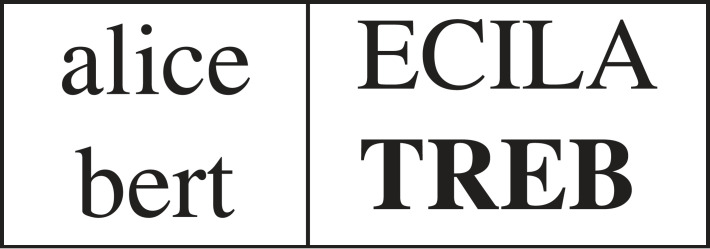
Expected response.

Consider a typical IQ-test question, based on textual analogy (see figure 1), which says that ‘alice’ is to ‘ECILA’ as ‘bert’ is to ‘?’. In this case assume the word indicated by ‘?’ is ‘TREB’ (figure 2) since the relation R between ‘alice’ and ‘ECILA’ is ‘the reverse of the uppercase of the given letter sequence’. If there are no limits on the length of the letter sequences, an infinity of alternative answers might have been possible, including ‘ECILA’. But for such an IQ-test question to be effective, the answer ‘TREB’ must have high consensus among typical participants. In this paper, it will be shown that standard computational learning theory (CoLT) [1,2] approaches do not account for humans’ ability to hypothesize such a relation, R, from a single example.

Question: How can we model human abilities to hypothesize R from a single example?

An answer to this question has potential to provide insights into human perception and reasoning, and could help to identify new methods for efficient human–machine interaction. One relevant point worth noting is that human subjects presented with the problem are likely to already know the relevant commonly understood sub-concepts of *uppercase* and *reverse sequence*. However, it is unclear how a person could identify these two relations as the most relevant out of a large number of candidate relations which they already know.

To address the question above a modified version of an existing CoLT approach [3] is developed. In the process, it is shown that for high expected-accuracy (EA) to be achieved on unseen cases, the Bayes’ optimal hypothesis selected needs to provide a trade-off between the description length and generality of the algorithm representing R. Additionally, in the case of learning from a single example it is shown that the algorithm being learned needs to have low generality, meaning the chances are low for it correctly predicting the truth value of a randomly selected instance. This model of highly data efficient concept learning has ramifications concerning the lifetime learning of concepts, such as R, by humans having access to an accumulated mental library of previously learned low-generality relations.

The paper is organized as follows. In §2, we review relevant work related to learning from one example, or *One-shot Learning*, in both Cognitive Science and Artificial Intelligence. Following this, §3 introduces a mathematical framework consisting of a learning protocol, a Bayes’ optimal solution and an associated expected-error (EE) bound for one-shot learning. Section 4 describes a new meta-interpretive learning (MIT) [4] system, called DeepLog, which supports both one-shot and few-shot learning of efficient algorithms from relational examples. DeepLog achieves this by identifying algorithms which combine low description length with low generality. DeepLog is used in the experiments described in §5, which indicate that high-accuracy algorithms can be efficiently learned from one example, not only for simple memory-free automata, but also for cases such as the textual analogy (figure 1), in which a push-down stack, of unbounded size, is required. In §6, we conclude and discuss possible areas for future research on this topic.

## 2. Related work

### (a) Cognitive science

Over the last two decades there have been an increasing number of papers (e.g. [5–9]) demonstrating machine learning algorithms which learn concepts from a single example. This is referred to as ‘one-shot learning’. Such algorithms are motivated by the observation that human learning often involves generalizations from a single example. However, such work has, to date, lacked mathematical analysis of the error associated with this form of learning. According to one of these papers published in the Cognitive Science literature [5] ‘People can learn ... concepts from just one example, but it remains a mystery how this is accomplished.’ Clarifying the elements of this mystery is the central motivation of the present paper.

### (b) Linguistics

As already pointed out in the example shown in figure 1, it is clear that some of the learning bias which enables one-shot learning comes from concepts already known to the human learner, such as *uppercase* and *reverse sequence*. However, it is not immediately obvious how many such background concepts humans have which might play a part in learning a new concept. One bound on available concepts comes from studies in linguistics on the typical size of human vocabulary. According to one such study [10] the average adult knows somewhere in the range of 10 000–42 000 words, including, in our ‘alice’ example case, ‘uppercase’ and ‘reverse’. However, any algorithm employing tens of thousands of background concepts would be not only overwhelmed by the available combinations of these terms, but also have to deal with the danger of over-fitting the example provided (e.g. always predicting ‘ECILA’). Despite this, humans are able to rapidly and reliably identify the relevance of ‘uppercase’ and ‘reverse’ and use these to hypothesize a new concept in the ‘alice/ECILA’ example.

### (c) Identification in the limit

**Figure 3.**
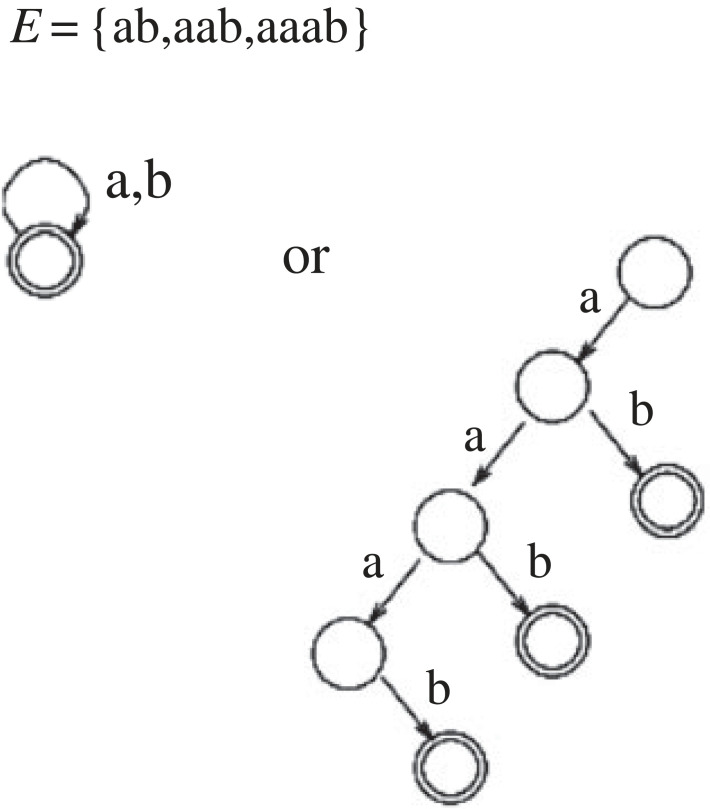
Identification in the limit. Complexity versus generality.

CoLT [11,12] is the study of algorithms which learn hypotheses from examples. The earliest such theoretical framework was introduced in 1967 by Gold [13], and is known as *Identification in the Limit*, in which learning algorithms which are provided with a finite enumeration of both hypotheses and examples of a target language, for positing consistent hypotheses. Learning is effective in the case there exists a finite prefix of the example sequence after which the learning algorithm chooses the target language, and does not subsequently alter its hypothesis. Gold proved that, in general, for infinite formal languages (such as regular and context-free languages in the Chomsky hierarchy) such identification is not possible when learning from positive examples alone. The reason, as shown in figure 3, is that for such language classes, given a finite number of examples, at no point can an algorithm discriminate between the most general language, consisting of all possible sequences from a given alphabet, or alternatively the most specific language, containing only the examples provided so far. Gold [13] pointed out the apparent disparity between this formal result and existing psycholinguistic studies by McNeill [14], which had shown that children learn language largely from positive examples, and tend to ignore corrections to their use of grammar.

### (d) Probably approximately correct learning

In 1984, a new framework for computational learning was introduced by Leslie Valiant [2]. By contrast to Gold’s requirement of exact identification, probably approximately correct (PAC) addresses learning algorithms which converge efficiently, with high probably, on an arbitrarily close approximation of the target theory, as increasing numbers of randomly selected examples are provided. Among those hypothesis classes considered by Valiant [2] only k-CNF propositional logic formulae, has a PAC convergence proof based on positive examples alone. However, for a given propositional signature a k-CNF formula has a finite domain, as opposed to the grammar classes with infinite domains studied by Gold. In general, positive learnability results for the PAC framework tend to be restricted to highly constrained hypothesis classes, and the upperbounds on out-of-sample error tend to be weak when compared to the actual error in experimental trials.

### (e) Bayesian positive-only learning

In 1996 [3], the author introduced a framework to analyse the learning of logic programs from positive-only examples.^1^ A Bayesian prior over the hypothesis space is assumed. The next section shows how a Bayesian approach allows the identification of maximal aposterior probably (MAP) hypotheses which provide a trade-off between the complexity of the hypothesis and its generality. It is shown that polynomial time logic programs can be learned with high accuracy from a randomly selected positive example sequence. This result goes beyond the positive-only results of Gold and Valiant, and supports efficient learning of infinite languages and programs. However, Muggleton [3] falls short of providing effective error bounds for one-shot learning. We will address this issue in the next section.

## 3. Theoretical framework

### (a) Bayesian learning protocol

For the purposes of this paper, we introduce a specialization of the teacher-learner positive-only protocol introduced in [3]. In this variant, relational instances are atoms r(a,b), where r is a relation (i.e. predicate of arity 2) and a and b are ground terms.^2^ A relational program is a logic program in which all predicates have arity 2.

— X is a countable^3^ set of relational instances.
— $D_{X}$ is the teacher’s probability distribution over X.
— $H\subset 2^{X}$ is a countable hypothesis set for which each H∈H represents the least Herbrand model of a relational program.
— $D_{H}$ is the teacher’s probability distribution over H.
— The teacher randomly chooses target theory T∈H from $D_{H}$ and then chooses $E=x_{1}\ldots x_{m}$ randomly and independently from $D_{X|T}$, with probability x∈E such that $D_{X|T}(x)=D_{X}(x|T)=D_{X}(x\cap T)/D_{X}(T)=\left\{ \begin{array}{ll} 0 & \text{if }x\notin T\\ \frac{D_{X}(x)}{D_{X}(T)} & \text{otherwise} \end{array}\right.$
— The learner now selects H∈H for which E⊆H.
— For H∈H, $D_{X}(H)=\sum_{x\in H}D_{X}(x)$.
— The teacher then assesses Error(H,T) as $D_{X}(H\setminus T)+D_{X}(T\setminus H)$.
— The EE of hypothesis H is then
  $$\mathrm{EE}(m)=\sum_{T\in H}D_{H}(T)\sum_{x\in E\in T^{m}}D_{X}(x|T)\mathrm{Error}(H,T).$$
  where the set of all cardinality m training sets $T^{m}$ is defined recursively as follows. $T^{1}=T$ and $T^{m}=T\times T^{m-1}$.
— $sz(H)=-\ln D_{H}(H)$ is referred to as the size of H.
— $g(H)=\sum_{x\in H}D_{X}(x)$ is referred to as the generality of H.

### (b) Positive-only MAP selection

**Figure 4.**
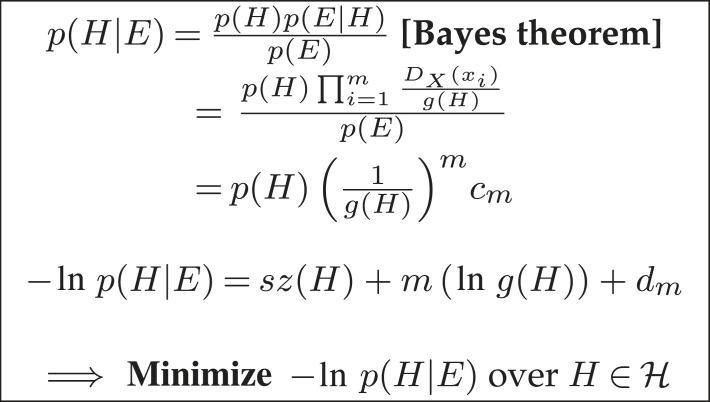
Positive-only MAP selection. m=|E|, $x_{i}\in E$, $c_{m}=\prod_{i=1}^{m}D_{X}(x_{i})$ and $d_{m}=\ln\;c_{m}$.

Using the Bayesian Learning Protocol the learner’s positive-only MAP selection method introduced in [3] is shown in figure 4. The complexity versus generality problem highlighted by Gold’s result (figure 3) is resolved by the learner selecting an hypothesis H which trades-off sz(H) and g(H).^4^ As the number of training examples m increases, the need to minimize g(H) progressively dominates minimizing sz(H), which contrasts with the fixed hypothesis ordering assumption of Gold [13]. Let us now consider Bayesian one-shot learning.

### (c) Bayesian one-shot learning

**Figure 5.**
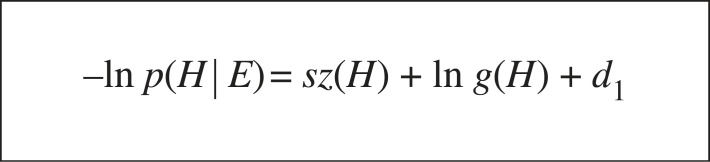
Bayesian one-shot learning, m=1 case.

One-shot learning is simply the special case of Bayesian positive-only learning for which m=1 (see figure 5). However, this case was not considered in [3], since the EE bound derived in that paper gives EE(1)≤2.33, which is trivially true since error is in the [0,1] interval.

### (d) Expected-error bounds

**Figure 6.**
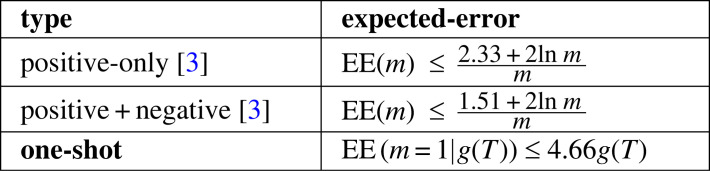
Previous and new expected-error bounds.

EE results are shown in figure 6. The positive-only result was based on the assumption that g(T)≤1/2. Under this assumption, it was noted [3] that the bounds indicate (see figure 6) that it does not take many more examples to learn from positive-only than from a mixture of positive and negative examples. This was found to be consistent with out-of-sample results for an implementation of these two approaches [3]. The third is a new result of the present paper. This can be derived by generalizing the g(T)≤1/2 assumption to g(T)≤α where 0≤α≤1, in which case EE(m)≤α(4.66+4ln m)/m. In the one-shot learning case, m=1, we get EE(m=1)≤α(4.66+4ln 1)/1 and therefore EE(m=1|g(T))≤4.66g(T).

### (e) Low-generality targets

**Figure 7.**
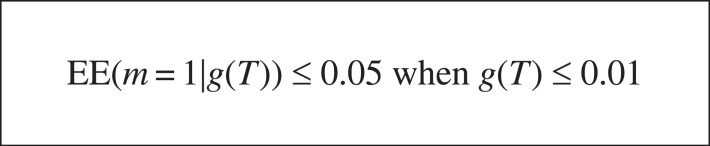
Low-generality/high-accuracy region.

The new one-shot EE bound in figure 6 allows us to predict high-accuracy hypotheses are possible from one example if and only if the generality of the target hypothesis is low (see figure 7).

### (f) Choice of representation

**Figure 8.**
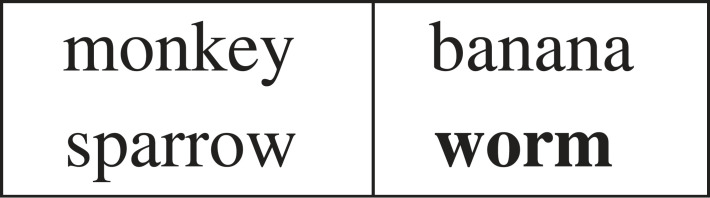
Analogy involving relation *eats*.

The motivation for this work is to analyse and demonstrate a general approach to learning from a single example in a way that models human abilities. One-shot learning of an algorithm is particularly challenging, since it involves a countably infinite domain (e.g. reversing and uppercasing a sequence such as ⟨a,l,i,c,e⟩). However, humans also have a capacity to identify relations from a single example, as part of a broad range of analogy problems. For instance, the answer ‘Insect’ to the analogy problem in figure 8 would be equally acceptable for a human to that given. For this reason, rather than select a functional programming language, the base representation is that of relational logic programs rather than functional programs, though the results in figure 6 would apply in either case since functions can be considered as many-to-one relations. In the following two sections, we will investigate the learning of relational logic programs from a single example, in order to test the one-shot result of figure 6 in practice.^5^

## 4. DeepLog implementation

### (a) DeepLog system

**Figure 9.**
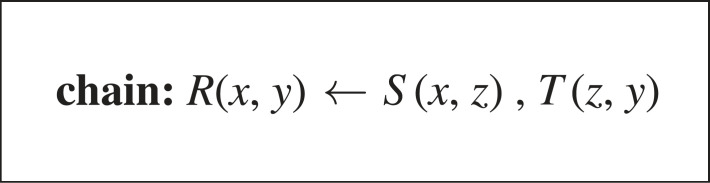
Chain metarule defines relation R as composition of S and T. Only clauses of this form are included in programs hypothesized by DeepLog.

DeepLog^6^^7^ is a new inductive logic programming (ILP) [15–17] system, implemented in SWI-Prolog, based on MIL [4,18–21]. ILP systems hypothesize a relational logic program H from background knowledge B, primitive relations library $B_{0}\subseteq B$, positive examples $E^{+}$ and negative examples $E^{-}$. MIL systems additionally require the user to provide a set of metarules M which indicate the structure of rules to be learned. By contrast, DeepLog minimizes the requirements on the user by providing background knowledge from an existing library of definitions and uses a fixed metarule set M consisting of only one of the metarules from the Metagol system [19], namely *Chain*, shown in figure 9. The chain rule allows the introduction of a relation R based on relational composition of S and T.

### (b) DeepLog architecture

**Figure 10.**
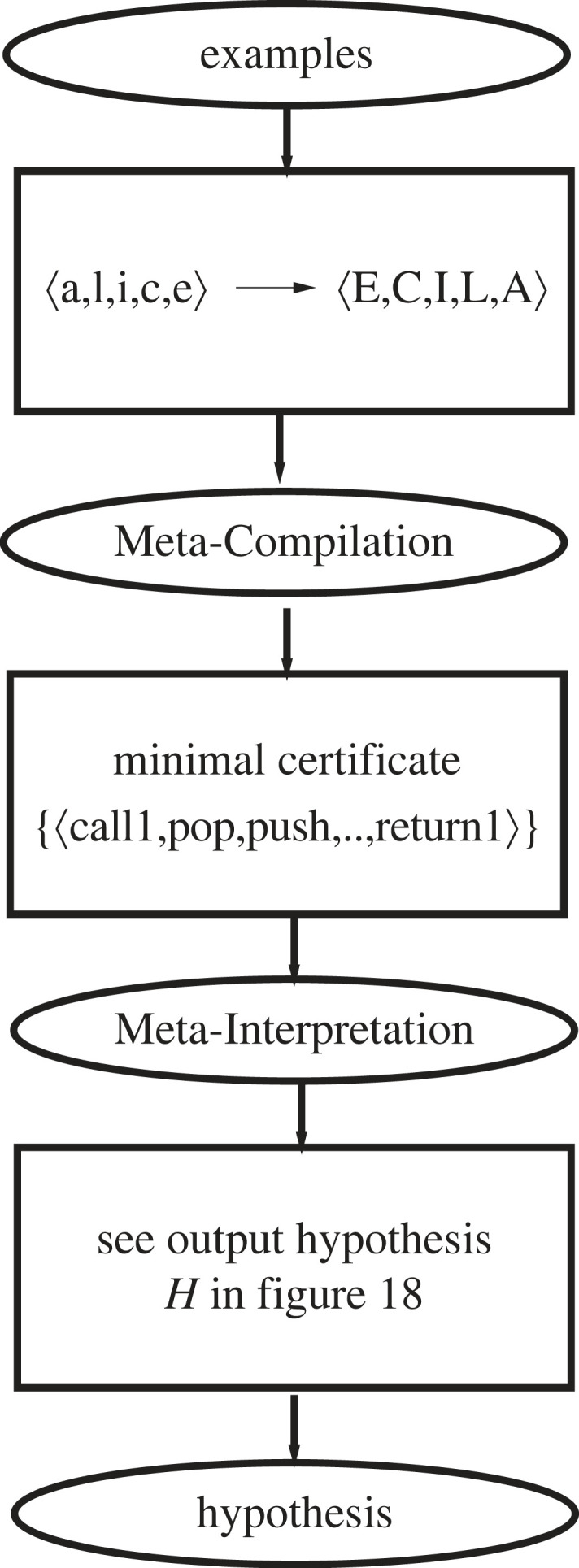
DeepLog stages.

The overall structure of DeepLog is shown in figure 10.

### (c) Meta-compilation

The positive examples $E^{+}$ are used to find a set of minimal length input–output certificates,^8^ where C is a length n certificate of an example r(a,b)∈E+ if r(a,b) is a relational instance, a and b are the logical terms and there exists a sequence of ground instances $C=\langle p_{0}(a,x_{0}),\ldots ,$ $p_{n}(x_{n},b)\rangle$ (simplified below to $C=\langle p_{0},\ldots ,$ $p_{n}\rangle$). $B_{0}⊨\alpha$ for each ground atom α in C. C has minimal length n in case there is no certificate $C^{\prime }$ for r(a,b) of length $n^{\prime }$ where $n^{\prime }<n$. This step is used to identify both a minimal signature of primitive relations from the library $B_{0}$ and a minimal universe of logical terms $x_{i}$ to be considered as intermediate states in the certificates considered by the meta-interpretation stage. Minimal (*Min*^9^ ) [3] and maximal (*Max*^10^ ) bounds on the number of clauses in any hypothesized program are identified. Additionally, repeated subsequences of the minimal certificates are identified as potentially useful auxiliary primitives. Finally, a set of binary square matrices are computed to provide a look-up oracle for the meta-interpreter to rapidly identify minimal certificates and optimal choice points in the meta-interpreter’s derivation of hypotheses.

### (d) Meta-interpretation

**Figure 11.**
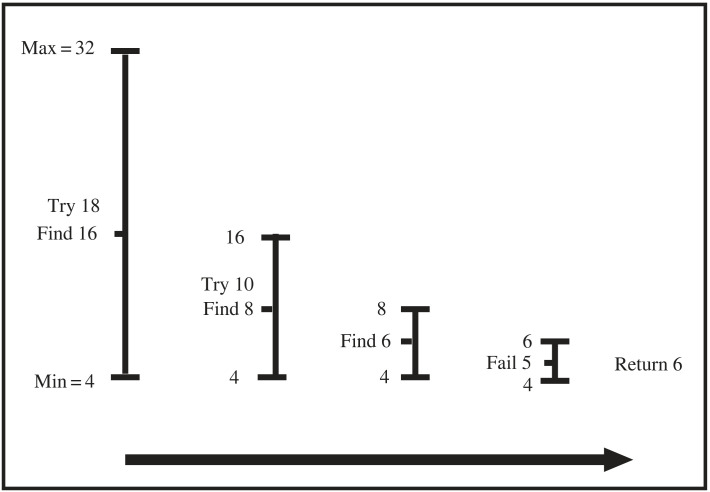
Binary search exponentially reduces hypothesis space to return consistent program with minimal number of clauses.

A binary search procedure (figure 11) is used to progressively reduce the ⟨Min, Max⟩ boundaries in order to find the minimum number of clauses in any consistent hypothesis returned by the meta-interpreter. For any specific ⟨Min, Max⟩ pair the meta-interpreter’s search order considers more specific hypotheses before more general ones, and returns the first consistent hypothesis. This strategy is aimed at finding low complexity hypotheses which have low generality, in accordance with the positive-only MAP selection strategy described in §3.

## 5. Experiments

In this section, the EE predictions from §3 are compared against the behaviour of DeepLog in the learning of three specific target programs. For each target program the same background library of 62 primitive relations is used. The library was developed progressively from investigating 57 different problems, and contains a set of type identifiers and basic relations over various types of entities, including characters, lists, stacks, numbers, three-dimensional positions, chess and family relations. In the problem descriptions below one-shot percentage EA is defined as EA(1,T)=100(1−EE(m=1|g(T))).

### (a) Problem1: regular grammar

**Figure 12.**
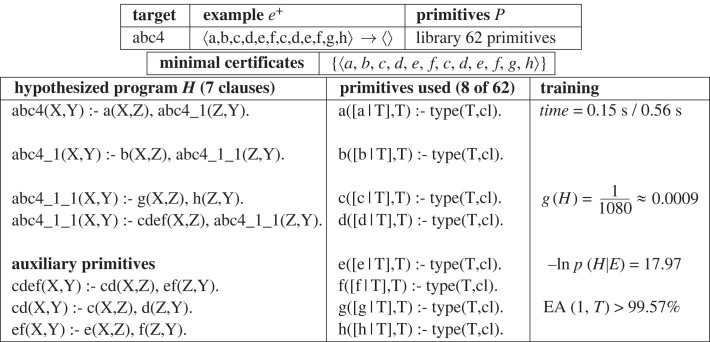
Problem1: Example, minimal certificate, hypothesis, primitives used and training. EA is defined as EA(1,T)=100(1−EE(H)). Primitives *a,b,…,h* remove a single character from the input to produce the output. The input and output type *cl* is a character list. The hypothesized program represents the regular grammar $ab{\left(cdef\right)}^{*}gh$

Consider a formal language which involves repeated letter sequences. For instance, the positive example sequence $e^{+}=\text{`abcdefcdefgh'}$ might be used to exemplify the target language $G=\text{ab}{\left(\text{cdef}\right)}^{*}\text{gh}$. G corresponds to letter sequences, such as $e^{+}$, which have a prefix ⟨a,b⟩, a suffix ⟨g,h⟩ and zero or more repetitions of ⟨c,d,e,f⟩ inbetween. Figure 12 shows $e^{+}$ as an input/output pair and DeepLog’s hypothesized Logic Program, corresponding to G. This program is constructed from primitives which reduce the sequence by one letter (such as a,b,…), auxiliary relations (such as cdef,cd,…) composed from the primitives introduced during Meta-Compilation (figure 10), and invented relations (such as abc4_1,abc4_1_1,…) introduced during Meta-Interpretation (figure 10).

Training time taken on a laptop (Intel-i7/2.80 GHz) is 0.15 s for meta-compilation and 0.56 s for meta-interpretation. The generality of the hypothesized program is g(H)≈0.0009, leading to an EA of EA(1,T)>99.57% and negative log posterior of 17.97. This corresponds to the low-generality criterion for one-shot learning, shown in figure 7.

**Figure 13.**
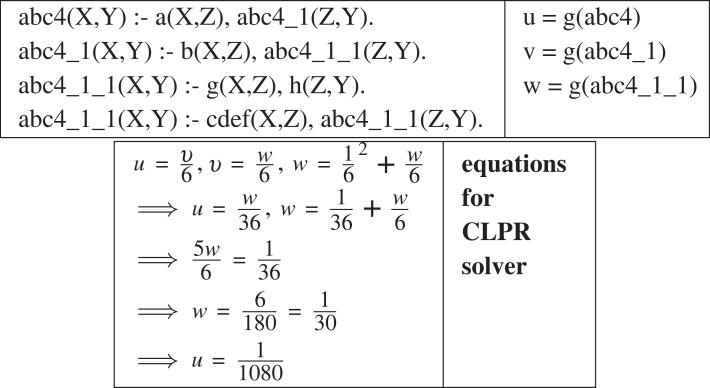
Calculation of g(H).

Figure 13 illustrates the way in which the value of g(H) is derived from equations using SWI-Prolog’s CLPR rational solver [22]. CLPR solves rational number equational constraints based on a polynomial-time solver. The equations used in figure 13 are derived by Deeplog from the definitions in the hypothesis. For instance, $w={\left(1/6\right)}^{2}+(w/6)$ is the sum of the generalities of the first and second clause of abc4_1_1.^11^ The generality of the first clause is $1/6^{2}$ since g(g)=g(h)=1/6 and g,h are selected from the six non-invented relations in the identified program. The generality of the second clause is w/6 since it is the product of g(abc_1_1) and g(cdef).

**Figure 14.**
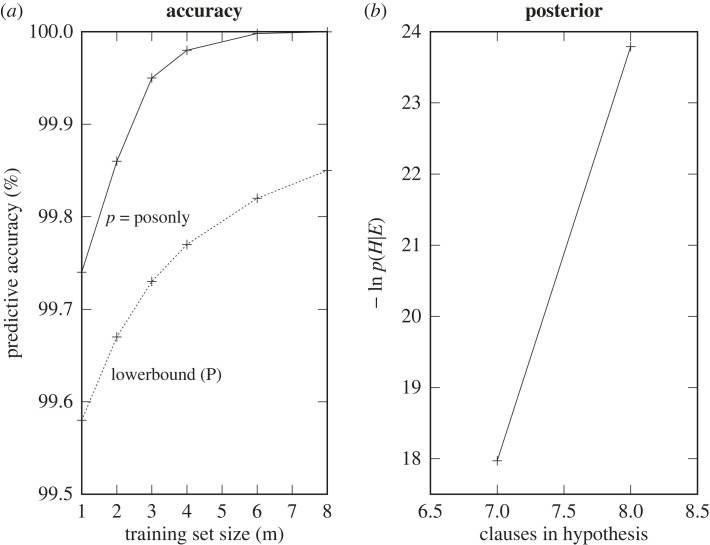
Problem1: (*a*) Accuracy increase and (*b*) negative log probability decrease.

Figure 14 shows (*a*) a comparison of Actual predictive accuracy for DeepLog versus the positive-only EE bound of figure 6 and (*b*) the variation of negative log posterior with decreasing clause bounds.

### (b) Problem2: Fire escape plan

**Figure 15.**
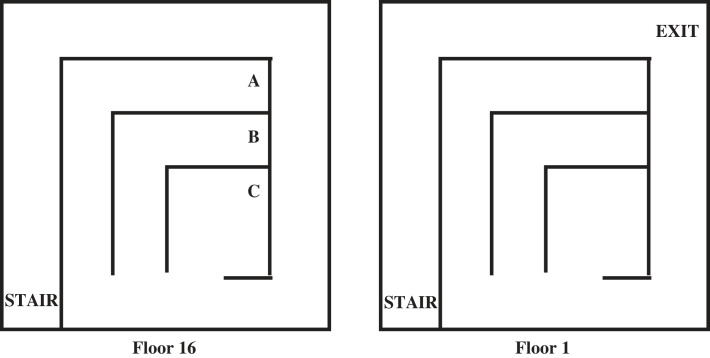
Problem2: Floorplan.

**Figure 16.**
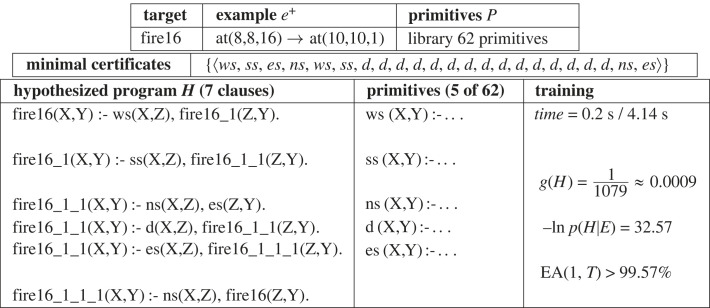
Problem2: Example, hypothesis and training. The example indicates that the agent starts at coordinate ⟨8,8,16⟩ position **A** (see figure 15) on floor 16 and needs to reach coordinate ⟨10,10,1⟩, position **Exit** on Floor 1. Primitives *ws, ss, ns* and *es* repeatedly move the agent in a given direction until obstructed, while primitive *d* moves the agent down one floor. While the Minimal Certificate represents an optimal plan for getting to the **Exit** from **A** on floor 16, the hypothesized program will get the agent to the **Exit** from **A**, **B** or **C** from any floor of the building.

**Figure 17.**
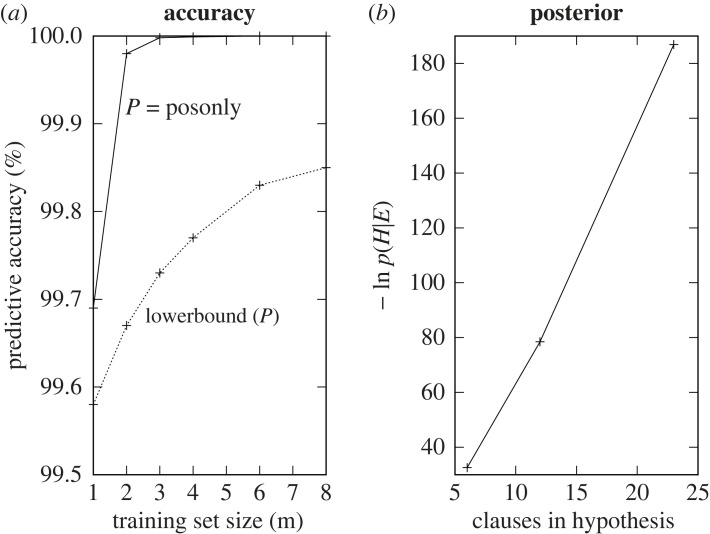
Problem2: (*a*) Accuracy increase and (*b*) negative log probability decrease.

This problem involves a general fire escape plan for leaving a 16 storey building. The floorplan is shown in figure 15. All floors have the same layout as Floor 16, except Floor 1, which contains the EXIT. Figures 16 and 17 show the problem description and results for the fire escape problem. Figure 17*a* shows once more that the learned program has low generality leading to high predictive accuracy from the first example provided, in accordance with the predictions of the positive-only EE bound in figure 6. Additionally, figure 17*b* indicates how two cycles of the Deeplog’s binary search process (see figure 11) successively reduce the number of clauses and negative log posterior of the hypothesis.

### (c) Problem3: Reverse uppercase

**Figure 18.**
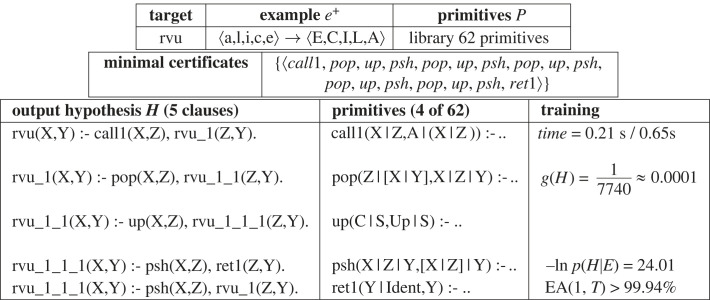
Problem3: Example, hypothesis and training. The primitive *call1* pushes an empty list *A* onto the program stack as an accumulator. Primitive *pop* removes the head of the input list and pushes it onto the program stack. Primitive *psh* replaces the top two elements X,Z of the program stack by a list with head *X* and tail *Y*. Primitive *up* replaces the letter at the top of the program stack by its uppercase version. Lastly, *ret1* replaces the program stack *Y—Ident* by the output list *Y*.

**Figure 19.**
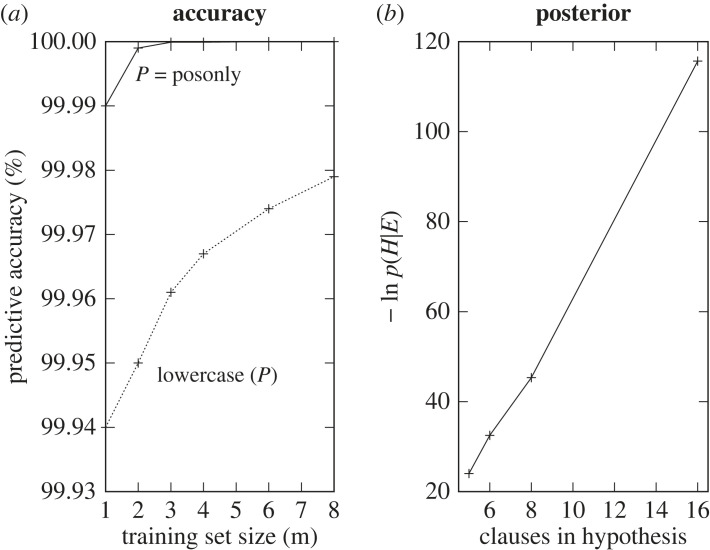
Problem3: (*a*) Accuracy increase and (*b*) negative log probability decrease.

We now consider the ‘alice’ to ‘ECILA’ textual analogy problem introduced in §1. This is shown in figure 18, which involves the construction of a function for reversing and making uppercase a letter sequence. Efficient Prolog programs normally require the introduction of an arity 3 auxiliary predicate to reverse a list. The same end is achieved with arity 2 predicates for DeepLog by dynamically creating a stack in the program state. Since Problem3 requires such a stack to be of unbounded size, the resulting program can be thought of as a push-down automaton (PDA) as opposed to the finite state automata (FSA) solutions in Problem1 and Problem2. PDAs represent a higher expressivity function class than FSAs since the stack can be treated as an unbounded Turing-machine tape. This additional functionality is enabled by the availability of the background library primitives *call1* (see figure 18), which introduces a new ‘empty’ object on the top of the calling stack. The type of the new object, in this case a list, is assigned by DeepLog using type-recognition predicates applied to the example provided. On exit this object is passed back using *ret1*. It should be noted that the resulting program would be relatively complex to encode manually, since it involves introduction of three subsidiary relations with mutual recursion and efficient interleaving of pushing, popping and uppercasing of letters. Training is achieved with EA from one example in under 1s on a laptop, and the generality and negative log probability are lower than in Problem1 and Problem2. This is reflected by more rapid accuracy and posterior convergence in figure 19*a*. Figure 19*b* shows that posterior converges is achieved in three binary search iterations (see figure 11) which reduce the hypothesis from 16 clauses down to the final 5 clause hypothesis returned.

## 6. Conclusion and further work

### (a) One-shot learning

The paper introduces a form of textual analogy problem as an example of human ability to hypothesize a concept effectively from a single example. This phenomenon, known as one-shot learning, has been extensively studied over the last decade within both Cognitive Science and Machine Learning. However, one-shot learning has not previously been explained within CoLT, and no theoretical framework exists for analysing its EE.

### (b) Bayes’ model of one-shot learning

A framework for analysing EE when learning from one example is introduced in this paper based on an adaptation of the author’s existing Bayesian analysis of positive-only learning. The approach enables, for the first time, an upper-bound error analysis of the human phenomenon of one-shot learning. EE bounds for one-shot learning are introduced as a special case of Bayesian positive-only learning. The analysis indicates that the effectiveness of one-shot learning is dependent on the target theory having generality below 0.01.

### (c) DeepLog experiments

A new system called DeepLog is introduced and its convergence properties are compared against the Bayesian EE bounds for one-shot and positive-only learning on three problems involving the conjecture of algorithms from one positive example. The error results for Deeplog are found to be consistent with the Bayes’ model error bounds.

### (d) One-shot learning in the context of science

Within the context of scientific discovery we suggest that one-shot hypotheses play the part of an initial conjecture, where subsequent examples provide either further confirmation, resulting in increased EA, or alternatively a refutation of the initial conjecture. Such initial conjectures, concerning observed phenomena, have played a vital role historically in the initial development of novel scientific theories, whether in positing Newtonian gravity or the theory of Mendelian genes. However, it seems reasonable to assume that such an ability is reliant on innate properties and abilities of human perception and reasoning.

### (e) Further work

The result in figure 7 indicates high-accuracy concepts can be learned with high data efficiency when the target has low generality. If learning is used to progressively accumulate low-generality background concepts, this should lead to a reduction in search combinations when learning new concepts for which the new concepts are relevant. Future work is needed to explore this effect.

For the sake of simplicity it has been assumed that examples are (i) noise-free and (ii) background primitives are correct and complete. Situations in which these assumptions do not hold should be explored in future work.

Finally, the EE bounds given in this paper and [3] fit empirical results more tightly than worst case bounds found in PAC learning. It might be possible to find even tighter bounds, though this is likely to come from making stronger assumptions than those found in [3].

## Data Availability

The data are provided in electronic supplementary material [23].

## Footnotes

1 Later Tenenbaum [8] investigated positive-only learning in a Bayesian framework and referenced [3] but concentrated on an approach based on finite extensions. Note that infinite extensions, such as those considered in [3] and §3 of this paper, are required for learning algorithms such as *reverse*.

2 For instance, X={rvu(⟨⟨A,l,i,c,e⟩,⟨E,C,I,L,A⟩⟩),rvu(⟨⟨B,e,r,t⟩,⟨T,R,E,B⟩⟩),…} are relational instances of the relation *rvu*. Note that an algorithm like reverse uppercase is usually defined over a countably infinite set, and is both a relation and a one-to-one function.

3 Either finite or countably infinite.

4 While [3] assumes $p(E|H)={\left(1/g(H)\right)}^{m}$, Tenenbaum’s *size principle* in [8] is that $p(E|H)={\left(1/\mathrm{size}(H)\right)}^{m}$. The former corresponds to the probability of H, with potentially infinite extension, being true of m instances randomly selected from $D_{X}$, while the latter is the non-normalized probability of concept H, with *finite extension* size (H), being true of m instances randomly selected from a uniform distribution over X.

5 Since this result applies to very low-generality hypotheses, finding a single satisfying solution is considered to be of more importance than the order in which multiple satisfying solutions should be presented.

6 ‘Deep’ refers to the initial deep dive to find a consistent certificate.

7 DeepLog code and datasets available at https://github.com/StephenMuggleton/DeepLog/.

8 In Computability theory these are called *certificates*, in Graph theory they are called *edge-labelled paths* and in the AI planning literature they are sequential plans.

9 *Min* is the length of the minimal number of relations in any minimal certificate identified.

10
*Max is one less than the length of a minimal certificate.*

11 The recursive definition of abc4_1_1 is reflected in the infinite sum $w={\left((1/6\right)}^{2}+(w/6))=({\left(1/6\right)}^{2}+{\left(1/6\right)}^{3}+{\left(1/6\right)}^{4}+\cdots )$ . CLPR efficiently solves sets of such interrelated equations to provide an exact rational solution.
